# High Rate of Initially Overlooked Kaplan Fiber Complex Injuries in Patients With Isolated Anterior Cruciate Ligament Injury

**DOI:** 10.1177/03635465211015682

**Published:** 2021-06-04

**Authors:** Daniel P. Berthold, Lukas Willinger, Matthew R. LeVasseur, Daniel E. Marrero, Ryan Bell, Lukas N. Muench, Zenon Kane, Andreas B. Imhoff, Elmar Herbst, Mark P. Cote, Robert A. Arciero, Cory M. Edgar

**Affiliations:** † Department of Orthopaedic Surgery, University of Connecticut, Farmington, Connecticut, USA; ‡ Department of Orthopaedic Sports Medicine, Technical University of Munich, Munich, Germany; § Department of Trauma, Hand and Reconstructive Surgery University Hospital, Münster, Germany; Investigation performed at the University of Connecticut, Farmington, Connecticut, USA

**Keywords:** anterior cruciate ligament, anterolateral rotatory instability, Kaplan fibers, iliotibial band, magnetic resonance imaging

## Abstract

**Background:**

Injuries to the Kaplan fiber complex (KFC) are not routinely assessed for in the anterior cruciate ligament (ACL)-deficient knee during preoperative magnetic resonance imaging (MRI). As injuries to the KFC lead to anterolateral rotatory instability (ALRI) in the ACL-deficient knee, preoperative detection of these injuries on MRI scans may help surgeons to individualize treatment and improve outcomes, as well as to reduce failure rates.

**Purpose:**

To retrospectively determine the rate of initially overlooked KFC injuries on routine MRI in knees with isolated primary ACL deficiency.

**Study Design:**

Case series; Level of evidence, 4.

**Methods:**

Patients who underwent isolated ACL reconstruction between August 2013 and December 2019 were identified. No patient had had Kaplan fiber (KF) injury identified on the initial reading of the MRI scan or at the time of surgery. Preoperative knee MRI scans (minimum 1.5 T) were reviewed and injuries to the proximal and distal KFs were recorded by 3 independent reviewers. KF length and distance to nearby anatomic landmarks (the lateral joint line and the lateral femoral epicondyle) were measured. Additional radiological findings, including bleeding, lateral femoral notch sign, and bone marrow edema (BME), were identified to detect correlations with KFC injury.

**Results:**

The intact KFC could reliably be identified by all 3 reviewers (85.9% agreement; Kappa, 0.716). Also, 53% to 56% of the patients with initially diagnosed isolated ACL ruptures showed initially overlooked injuries to the KFC. Injuries to the distal KFs were more frequent (48.1%, 53.8%, and 43.3% by the first, second, and third reviewers, respectively) than injuries to the proximal KFs (35.6%, 47.1%, and 45.2% by the first, second, and third reviewers, respectively). Bleeding in the lateral supracondylar region was associated with KFC injuries (*P* = .023). Additionally, there was a positive correlation between distal KF injuries and lateral tibial plateau BME (*P* = .035), but no associations were found with the lateral femoral notch sign or other patterns of BME, including pivot-shift BME.

**Conclusion:**

KF integrity and injury can be reliably detected on routine knee MRI scans. Also, 53% to 56% of the patients presenting with initially diagnosed isolated ACL ruptures had concomitant injuries to the KFC. This is of clinical relevance, as ACL injuries diagnosed by current routine MRI examination protocols may come with a high number of occult or hidden KFC injuries. As injuries to the KFC contribute to persistent ALRI, which may influence ACL graft failure or reoperation rates, significant improvements in preoperative diagnostic imaging are required to determine the exact injury pattern and to assist in surgical decision making.

The Kaplan fiber complex (KFC) consists of the proximal and distal Kaplan fibers (KFs), which connect the distal iliotibial band to the posterolateral femur, and has recently garnered increased research interest, especially in patients with anterior cruciate ligament (ACL) injuries.^4,18,29^ In particular, its role in providing anterolateral rotatory stability has been the subject of several biomechanical and clinical studies underlining the importance of an intact anterolateral complex to resist internal rotational torques, especially at higher degrees of knee flexion.^9,19,26^ Consequently, persistent anterolateral rotatory instability (ALRI), which has been shown to occur in 15% of cases after primary ACL reconstruction,^1,2^ has been found to be associated with inferior clinical results and subjective instability.^16,20,21^

Recent studies have described the location and appearance of the proximal and distal KFs on magnetic resonance imaging (MRI) scans in ACL-intact knees, with both Batty et al^3^ and Berthold et al^6^ emphasizing that improvements in preoperative diagnosis are required to determine the exact injury pattern. To date, data on concomitant KFC injuries in ACL-deficient knees remain limited,^4^ which may be related to the complexity of the anterolateral complex and the variations of the KF attachments to the femur. Recent literature suggests that the reported incidence of injuries to the KFC is inconsistent, between 3% and 33%; however, small patient cohorts may be a limitation of these studies.^3,4,6,18,29,33^

Currently, identifying KFs on MRI remains challenging. This could lead to a high number of neglected KFC injuries, which is of clinical relevance, as accurate and early identification of these injuries may avoid delayed treatment and facilitate surgical decision making for additional anterolateral procedures in an ACL-deficient knee. Lateral extra-articular tenodesis and anterolateral ligament reconstructions have been shown to restore knee kinematics in the setting of combined anterolateral complex and ACL injuries,^15^ with the indication for an additional anterolateral procedure still remaining a matter of debate.^9^

The purpose of this study was to retrospectively evaluate the rate of initially overlooked KFC injuries, including the proximal and distal KFs, in an isolated ACL-deficient knee by using routine MRI protocols. It was hypothesized that injuries to the KFC in the isolated ACL-deficient knee would be frequent.

## Methods

Following institutional review board approval at the University of Connecticut (No. 20X-119-1), a retrospective review was performed on a consecutive cohort of patients undergoing ACL reconstruction at the authors’ institution. All patient records were obtained from a single practice of a senior knee specialist (C.M.E.) from August 2013 to December 2019. Patients were eligible for inclusion if noted to have an ACL tear as initially diagnosed on MRI and confirmed arthroscopically at the time of surgery. No patient had had KF injury identified on the initial reading of the MRI scan or at the time of surgery. Only after performing our previous study on how to detect KFs^6^ did we know in which planes and sequences to look for these injuries. Patients were excluded if they had a history of previous surgery in the index knee or concomitant injuries such as a tear of the posterior cruciate ligament, grade 2 or 3 injury to the lateral collateral ligament or the medial collateral ligament, lateral or medial meniscal tears, second fracture, anterolateral ligament injury, or a fracture. Additionally, patients were excluded if they received MRI scans longer than 3 months after injury, as an increased interval between injury and MRI (>3 months) may lead to a higher number of missed injuries to the KFC.^4^ Patients with a low-resolution preoperative MRI scan, given by the presence of motion artifacts, inadequate magnetic field strength (minimum 1.5 T), slice thickness >4 mm, <3 planes, or poor resolution that precluded adequate visualization of the injured knee were also excluded.

A data collection sheet was used to record patients’ characteristics (age, sex, laterality, and body mass index), date of injury, and date of MRI. Clinical visit and operative notes were used to capture physical examination findings, including Lachman, anterior drawer, and pivot-shift tests.

### MR Imaging and Analysis

**Figure 1.**
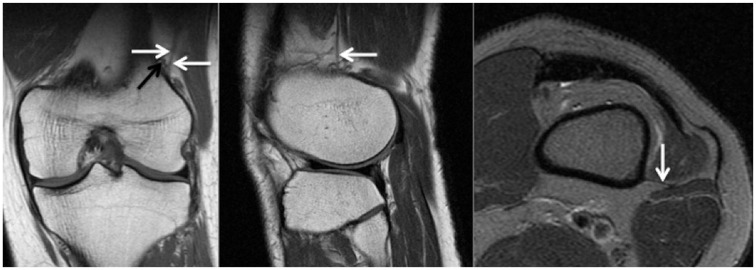
Coronal (left) and sagittal (middle) T1 MRI scans and axial (right) T2 fat-suppressed MRI scan of a left knee with intact KFs in all planes. The left image shows intact proximal and distal KFs (white arrows) and their proximity to the superior genicular artery (black arrow). KF, Kaplan fiber; MRI, magnetic resonance imaging.

**Figure 2.**
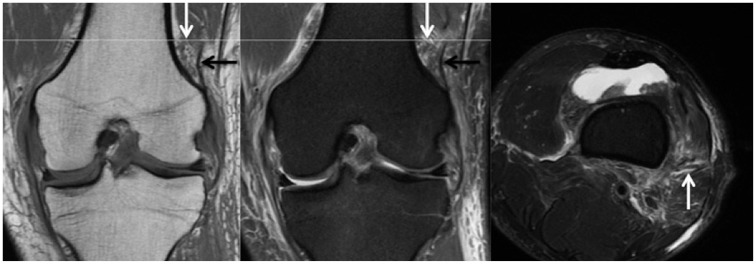
Matched coronal MRI scans (left: T1; middle: proton density fat-suppressed) of a left knee with corresponding axial plane level (right: T2 fat-suppressed MRI scan) demonstrating discontinuity of the proximal KF bundle (white arrow), with periligamentous edema but an intact, dark signal of the distal KF bundle (black arrow). KF, Kaplan fiber; MRI, magnetic resonance imaging.

**Figure 3.**
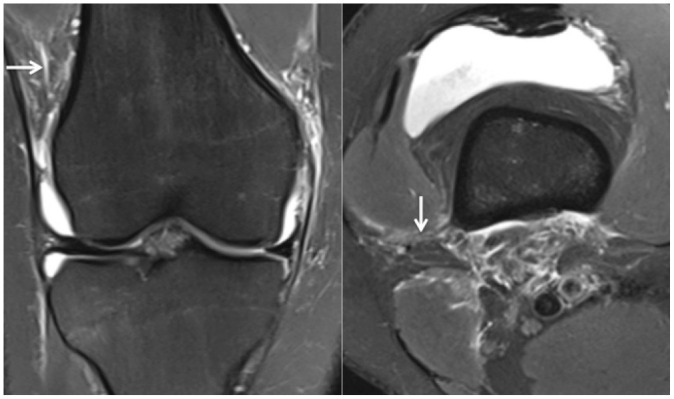
Coronal (left) and axial (right) T2 fat-suppressed MRI scans of a right knee demonstrating KFC injury. Both images are representative of distal KF injury (white arrows). KF, Kaplan fiber; KFC, Kaplan fiber complex; MRI, magnetic resonance imaging.

**Figure 4.**
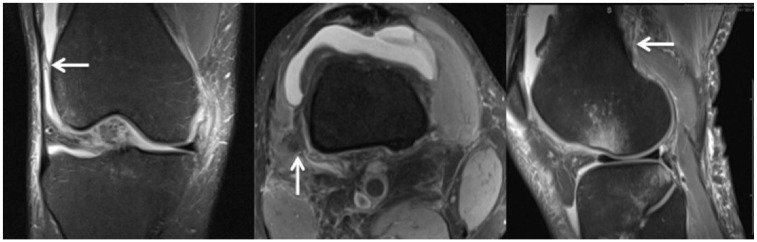
Coronal (left), axial (middle), and sagittal (right) T2 fat-suppressed MRI scans of a right knee demonstrating KFC injury (arrows). All images represent interstitial tears and periligamentous edema of the KFs. The coronal image is representative of distal KF injury and shows an adjacent effusion that aids in detection. The sagittal image shows the pivot-shift BME pattern to the posterolateral tibial plateau in conjunction with the anterolateral femur. BME, bone marrow edema; KF, Kaplan fiber; KFC, Kaplan fiber complex; MRI, magnetic resonance imaging.

MR images were analyzed independently by 2 fellowship-trained orthopaedic surgeons (D.P.B. and L.W.) and a musculoskeletal radiologist (D.E.M.) using the institution’s electronic picture archiving and communication system (Philips Corp). All scans were reviewed in all 3 planes (axial, coronal, and sagittal) as previously described.^3,6^ The integrity of the KFC was assessed according to Van Dyck et al^29^ and Khanna et al.^18^ KFs were classified as intact if low signal intensity fibers were seen attaching to the femur.^29^ Partial rupture was defined by an altered signal within the ligament, periligamentous edema, and disruption of the fibers.^18^ Complete tears were defined as complete disruption of the KFs.^29^ However, it was observed that routine preoperative knee MRI scans did not allow differentiation of partial or complete injuries. As such, partial or complete injuries were collectively identified as injuries to the KFC. Figure 1 shows a patient with an intact KFC and delineation of proximal and distal bundles. Figures 2 and 3 show proximal and distal KFs injuries, respectively. Figure 4 demonstrates a KFC injury with associated pivot-shift bone marrow edema (BME) changes, postinjury effusion, and hemorrhage, which can facilitate the detection of KFs.

Lengths of the KFs and distance to nearby anatomic landmarks, namely, the lateral joint line and the lateral femoral epicondyle, were measured using methods developed a priori.^6^ The lengths of the proximal and distal KFs from the distal femoral metaphysis to the iliotibial band were measured on the axial (T1) MRI scan. The distances from the metaphyseal attachment of the KFs to the lateral joint line and the lateral femoral epicondyle were measured on the coronal (T1) MRI scan.

Associated secondary radiographic markers, such as bone bruise (according to Brittberg and Winalski^8^) and presence of a lateral femoral notch sign (according to Warren^30^), as a marker for a high-grade pivot shift, were also evaluated on T2 fat-suppressed slices and T1 slices,^5^ respectively. BME was identified at the medial and lateral knee joint on both the tibial and femoral sides. As described by Khanna et al,^18^ and in accordance with Batty et al,^4^ pivot-shift BME pattern was also identified. A depth of 2 mm was selected as the cutoff for a pathognomonic lateral femoral notch sign, as previously described.^13^ Additionally, bleeding of the superior lateral genicular artery was recorded as well. KFs have been shown to lie in close proximity to the superior genicular artery, and injury can show MRI findings suggestive of hemorrhage.^3,6,10,12^

### Data Analysis

The frequency of classifying the integrity of KFs as intact or injured, for both the complex collectively or individually, was recorded for the 3 independent observers (D.P.B., L.W., D.E.M.). A 2-way random interclass correlation coefficient (ICC) (ICC, 2,1) was used to assess the interobserver reliability of the measurements of the KFs. ICC values were calculated for absolute agreement and consistency of agreement. ICC values were graded as follows: <0.4 poor reliability, 0.4-0.75 moderate reliability, and >0.75 excellent reliability.^27^ The length of the KFs and the distance of their femoral attachments to the lateral joint line and to the lateral femoral epicondyle were presented as means of the measurements from all reviewers. Continuous variables are given as means ± SD, with categorical variables being presented as numbers (percentages). To analyze for potential associations between identified KFC injuries and other radiographic findings (bleeding, lateral femoral notch sign, and BME changes), the chi-square test or the Fisher exact test were used where appropriate. KFs were considered injured if the proximal or distal part was ruptured and were considered intact if both were intact. All analyses were performed using Stata statistical software (StataCorp).

## Results

**Figure 5.**
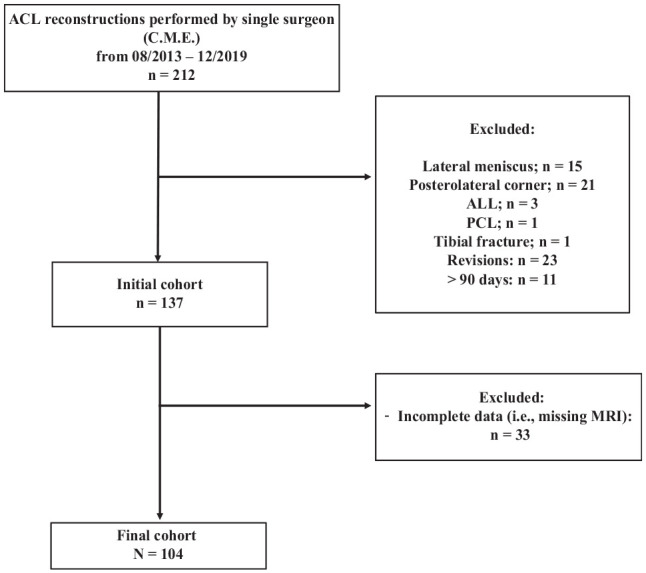
Flowchart representing the final cohort after applying inclusion and exclusion criteria. ACL, anterior cruciate ligament; ALL, anterolateral ligament; MRI, magnetic resonance imaging; PCL, posterior cruciate ligament.

In a single surgeon’s practice (C.M.E.), 212 patients underwent ACL reconstruction between August 2013 and December 2019. Of those, 104 patients were eligible for inclusion in the study (Figure 5). The mean age of the patients was 26.8 ± 10.7 years (range, 13-57 years), with the study population comprising 49 men and 55 women. Additional data on patients’ characteristics are delineated in Table 1.

**Table 1:** Patient Characteristics*^a^* (N = 104)

	Value
Age, y, mean ± SD (range)	26.8 ± 10.7 (13-57)
Body mass index, mean ± SD (range)	27.3 ± 5.6 (17.8-50.6)
Sex, n (%)	
Male	49 (47.1)
Female	55 (52.8)
Laterality, n (%)	
Right	53 (50.9)
Left	51 (49.0)

a BMI, body mass index.

### KF Identification for Presence and Integrity

Overall, injuries to the KFC were identified in 52.9% to 55.8% of cases. The first reviewer identified proximal and distal KFs injuries in 35.6% (n = 37) and 48.1% (n = 50) of cases, respectively, compared with 47.1% (n = 49) and 53.8% (n = 56) by the second reviewer, and 45.2% (n = 47) and 43.3% (n = 45) by the third reviewer, respectively. In addition, distal KF injuries were identified more frequently than injuries to the proximal KFs. Overall, there were excellent interrater agreements with respect to classifying the KFC as intact or injured, with the Kappa value of 0.716 and agreement in 85.9% of cases. With respect to proximal and distal KFs, there was less agreement (79.5%) with reporting the integrity of the proximal KFs (Kappa, 0.581). Classification frequency and percentages of all reviewers and Kappa values for interrater agreements are shown in Table 2.

**Table 2:** Assessment of KFC and Individual Component Integrity*^a^*

	Reviewer 1	Reviewer 2	Reviewer 3	Interrater
KFC				
Intact	49 (47.1)	46 (44.2)	49 (47.1)	Agreement: 85.9
Defect	55 (52.9)	58 (55.8)	55 (52.9)	Kappa: 0.716
				SE: 0.054
PKF				
Intact	67 (64.4)	55 (52.9)	57 (54.8)	Agreement: 79.5
Defect	37 (35.6)	49 (47.1)	47 (45.2)	Kappa: 0.581
				SE: 0.061
DKF				
Intact	54 (51.9)	48 (46.2)	59 (56.7)	Agreement: 82.1
Defect	50 (48.1)	56 (53.8)	45 (43.3)	Kappa: 0.641
				SE: 0.058

a Data are provided as n (%). DKF, distal Kaplan fibers; KFC, Kaplan fiber complex; PKF, proximal Kaplan fibers.

### KF Measurements

Measurements of the KF length and the distance from nearby anatomic landmarks assist with confirmation of KF identification at the distal femoral metaphysis. Based on the axial MRI plane, the lengths of the proximal and distal KFs were identified as 20.8 mm and 15.1 mm, respectively. Based on the coronal MRI plane, the distance of the proximal and distal KF attachments to the lateral joint line was 57.6 mm and 44.9 mm, and the distance to the lateral femoral epicondyle was 31.6 mm and 18.0 mm, respectively. These results are shown in Table 3.

**Table 3:** KF Lengths and Respective Distances From the Lateral Joint Line and Lateral Femoral Epicondyle*^a^*

	Length, mm		Distance to Lateral Joint Line, mm		Distance to Femoral Epicondyle, mm	
	PKF	DKF	PKF	DKF	PKF	DKF
Mean	20.8	15.1	57.6	44.9	31.6	18
SD	3.1	3	6	5.6	5.2	4.6
Minimum	15.1	5.9	41	30.1	19.1	1.3
Maximum	28.2	20	70.9	57.7	43.6	26.4

a DKF, distal Kaplan fibers; KF, Kaplan fiber; PKF, proximal Kaplan fibers.

### KF Injury and Associations to Other Radiographic Signs

The frequency of bleeding, lateral femoral notch sign, and BME changes, and their respective correlations with respect to KF injury are shown in Table 4. The MRI evidence of bleeding in the lateral supracondylar region was significantly correlated with KFC injury (*P* = .023). Additionally, there was a statistically significant correlation between the lateral tibial plateau BME and distal KF injury (*P* = .035). There was no association with other BME changes, including the pivot-shift BME pattern, or the lateral femoral notch sign with respect to the entire complex or individual KF bundles.

**Table 4:** Associations of KF Injury and Other Radiographic Sign Measurements*^a^*

	Frequency*^b^* (N = 104)	KFC*^c^* (n = 55)	PKF*^c^* (n = 37)	DKF*^c^* (n = 50)
Bleeding	44 (42.3)			
Defect		29 (52.7)	20 (54)	20 (50)
*P* value		.023	ns	ns
Lateral femoral notch sign	24 (23.1)			
Defect		12 (21.8)	9 (24.3)	12 (24)
* P* value		ns	ns	ns
Bone bruise lateral femur	49 (47.1)			
Defect		27 (49.1)	16 (43.2)	25 (50)
* P* value		ns	ns	ns
Bone bruise medial femur	16 (15.4)			
Defect		9 (16.4)	5 (13.5)	9 (18)
* P* value		ns	ns	ns
Bone bruise lateral tibia	64 (61.5)			
Defect		38 (69)	23 (62.1)	36 (72)
* P* value		ns	ns	.035
Bone bruise medial tibia	28 (26.9)			
Defect		16 (29.1)	9 (24.3)	15 (30)
* P* value		ns	ns	ns
Pivot shift BME	44 (42.3)			
Defect		25 (45.5)	14 (37.8)	24 (48)
*P* value		ns	ns	ns

a BME, bone marrow edema; DKF, distal Kaplan fibers; KF, Kaplan fiber; KFC, Kaplan fiber complex; ns, not significant; PKF, proximal Kaplan fibers.

b Percentages are given with respect to the final patient cohort (N = 104).

c Percentages are given with respect to the number of patients with the listed KF injury.

## Discussion

The most important finding of this study was that the presence and integrity of the KFC could be reliably detected on a routine MRI scan with excellent interrater agreement. Based on the data gathered from this retrospective study, more than 50% of the patients presenting with initially diagnosed isolated ACL injuries had concomitant injuries to the KFC. This is of clinical relevance, as injuries to the KFC contribute to ALRI and may have significant clinical implications on ACL graft failure or reoperation rates. Graft rupture of 28% has been reported in high-risk populations and only 50% to 65% of recreational athletes are able to return to their preinjury level of sports.^14,22,31,32^ These high percentages emphasize that peripheral lesions, such as injuries to the anterolateral complex, including the KFC, and subsequent rotatory instability may often be overlooked on preoperative MRI scans. As a result, the surgeon’s choice whether to perform isolated ACL reconstruction or additional lateral extraarticular tenodesis may be significantly influenced. Thus, significant improvements in preoperative diagnostic criteria are required to determine the exact injury pattern and assist in surgical decision making.^6^

As clinically relevant ALRI has been shown to occur in approximately 15% of cases after primary ACL reconstruction,^1,2^ several biomechanical and clinical studies have extensively studied the anterolateral complex and its role in resisting internal rotational torques.^7,9,10,19,23-25,28^ Kittl et al^19^ demonstrated that the iliotibial band and its deep structures incorporating the KFC are primary stabilizers against tibial internal rotation, mostly in ACL-deficient knees. As the proximal and distal KFs connect the deep iliotibial band to the distal posterolateral femoral metaphysis, high-grade injury may result in significantly increased ALRI.^9,11,12^ However, clinical diagnosis of persistent ALRI remains highly challenging, even for experienced surgeons.

Recently, Batty et al^3^ and Berthold et al^6^ emphasized the importance of improving preoperative diagnostics to detect injuries of the KFC more reliably, with the goal of substantiating the indications for lateral extraarticular tenodesis procedures in the setting of persistent ALRI. On the basis of previous anatomic descriptions by Kaplan^17^ and Godin et al,^10^ Batty et al^3^ demonstrated that the KFs and their proximal and distal attachments can be detected on MRI scans in ACL-intact knees. By using 3T MRI with a slice thickness of 3 mm, the authors showed that visualization of the KFs remains difficult, with high variability in interpretation and moderate interrater reliability for identification. Subsequently, Berthold et al^6^ demonstrated reliable visualization of KFs on MRI scans by performing a subsequent anatomic dissection of the same specimen. In contrast to Batty et al,^3^ the authors used a 3D T1-weighted and a T2-weighted turbo spin echo sequence, which could be implemented in routine protocols.^6^ Additionally, both studies were conducted on ACL-intact knees, which brings into question how an injured KFC would appear on MRI scans in an ACL-deficient knee.^3,6^

Recently, several studies have attempted to quantify the prevalence of KFs injuries in the setting of ACL injuries.^4,18,29^ Khanna et al^18^ described the KFs as “proximal” and “epicondylar” bands attaching to the posterolateral femur and showed that increased signal and thickening around the proximal and epicondylar bands were seen in 82% and 29% of ACL-deficient knees, respectively, in the setting of positive pivot-shift BME changes. Van Dyck et al^29^ showed that tears of the KFs were seen in 33% of knees with ACL injuries, with only 3% being classified as grade II or III.^29^ However, the low incidence may be limited to the small sample size of the study. More recently, Batty et al^4^ identified KF injuries in 18.6% of 161 patients who had preoperative MRI scans for ACL injury. This is in contrast to the current study, as all reviewers identified injuries to the KFC in more than 50% of cases. However, these differences may be because Batty et al^4^ included MRI scans taken more than 3 months after sustaining the ACL injury, which could lead to a high percentage of missed injuries. Furthermore, the diagnostic criteria proposed by Batty et al^4^ were noted to be more stringent compared with those by Khanna et al and this study, which may explain the low reported rates of injury to the KFC.

In the present study, the only associated radiographic marker with a statistically significant correlation suggestive of KFC injury was bleeding in the lateral femoral supracondylar region and for distal KFs injury was the lateral tibial plateau BME. A positive correlation with bleeding and KFC injury seems logical, as the proximity of the superior genicular artery to KF has been consistently established in the literature.^3,10,12^

There are several limitations to the study. First, this was a retrospective study design, which may lead to a selection bias. Second, this was a radiographic study without correlation with clinical and functional outcomes. Third, the included MRI scans were not performed according to the same protocol, and at times, the most proximal extent of the KFC was outside the field of view.^4^ However, this reflects daily clinical practice, as not all MRI protocols include a 3D reconstruction or are performed at the same radiology suite. As arthroscopic confirmation of ACL injury was required for inclusion in the study, nonoperative cases were not included, which could have implications for the prevalence of KF injury in knees with ACL tears. Fifth, there was not an intraoperative macroscopic assessment on the integrity of the KFs. Finally, there were inherent limitations in the quality of the MRI scans to visualize these smaller structures. Again, this deficiency emphasizes the need for accurate MRI protocols in the setting of acute ACL injuries and concomitant ALRI. Last, by limiting the time frame for MRI to <3 months, one may think that the natural history of injuries to the KFs may be missed, as this structure may have a potential to heal on its own, given its extraarticular anatomy. In our experience, by limiting the time frame for MRI to only 3 months, the authors intended to make sure that the natural history of KFs injuries were not missed, as these injuries mostly occur with ACL injury. Additionally, the authors were unsure if injuries of the KFs “heal” on their own, as none of the reviewers was able to identify scar tissue or similar in knees with KFC disruption. As this might be outside of the scope for this paper, the healing potential of torn KFs could be of interest for future investigations.

## Conclusion

KF integrity and injury can be reliably detected on a routine knee MRI scan. Also, 53% to 56% of the patients presenting with initially diagnosed isolated ACL ruptures had concomitant initially overlooked injuries to the KFC. This is of clinical relevance, as the current routine MRI protocol reveals a high number of occult or hidden KFC injuries. As injuries to the KFC contribute to persistent ALRI, which may influence ACL failure or reoperation rates, significant improvements in preoperative diagnostic criteria are required to determine the exact injury pattern and to assist in surgical decision making.
